# Radiological assessment of dementia: the Italian inter-society consensus for a practical and clinically oriented guide to image acquisition, evaluation, and reporting

**DOI:** 10.1007/s11547-022-01534-0

**Published:** 2022-09-07

**Authors:** Francesca B. Pizzini, Enrico Conti, Angelo Bianchetti, Alessandra Splendiani, Domenico Fusco, Ferdinando Caranci, Alessandro Bozzao, Francesco Landi, Nicoletta Gandolfo, Lisa Farina, Vittorio Miele, Marco Trabucchi, Giovanni B. Frisoni, Stefano Bastianello

**Affiliations:** 1 Radiology, Department of Diagnostic and Public Health, University of Verona, Piazzale L.A. Scuro, 10, 37100 Verona, Italy; 2 Department of Neurosciences, Biomedicine and Movement Sciences, University of Verona, Verona, Italy; 3 Department of Medicine and Rehabilitation, Clinical Institute S. Anna-Gruppo San Donato, Brescia, Italy; 4 Italian Society of Gerontology and Geriatrics (SIGG), Florence, Italy; 5 Italian Association of Psychogeriatrics (AIP), Brescia, Italy; 6 Department of Biotechnological and Applied Clinical Sciences, University of L’Aquila, L’Aquila, Italy; 7 Foundation Policlinico Universitario A. Gemelli IRCCS, Rome, Italy; 8 Department of Medicine of Precision, School of Medicine, “Luigi Vanvitelli” University of Campania, 80147 Naples, Italy; 9 NESMOS, Department of Neuroradiology, S. Andrea Hospital, University Sapienza, Rome, Italy; 10 Diagnostic Imaging Department, Villa Scassi Hospital-ASL 3, Corso Scassi 1, Genoa, Italy; 11 Neuroradiology Department, IRCCS Mondino Foundation, Pavia, Italy; 12 Dipartimento Di Radiodiagnostica Emergenza-Urgenza, Azienda Universitaria Careggi, Florence, Italy; 13 University of “Tor Vergata”, Rome, Italy; 14 Centre de La Mémoire, Geneva University and University Hospitals, 1205 Geneva, Switzerland; 15 Department of Brain and Behavioral Sciences, University of Pavia, Pavia, Italy

**Keywords:** Dementia, Consensus, Neuroimaging, MRI, Assessment

## Abstract

**Background:**

Radiological evaluation of dementia is expected to increase more and more in routine practice due to both the primary role of neuroimaging in the diagnostic pathway and the increasing incidence of the disease. Despite this, radiologists often do not follow a disease-oriented approach to image interpretation, for several reasons, leading to reports of limited value to clinicians. In our work, through an intersocietal consensus on the main mandatory knowledge about dementia, we proposed a disease-oriented protocol to optimize and standardize the acquisition/evaluation/interpretation and reporting of radiological images. Our main purpose is to provide a practical guideline for the radiologist to help increase the effectiveness of interdisciplinary dialogue and diagnostic accuracy in daily practice.

**Results:**

We defined key clinical and imaging features of the dementias (A), recommended MRI protocol (B), proposed a disease-oriented imaging evaluation and interpretation (C) and report (D) with a glimpse to future avenues (E). The proposed radiological practice is to systematically evaluate and score atrophy, white matter changes, microbleeds, small vessel disease, consider the use of quantitative measures using commercial software tools critically, and adopt a structured disease-oriented report.

**Summary statement:**

In the expanding field of cognitive disorders, the only effective assessment approach is the standardized disease-oriented one, which includes a multidisciplinary integration of the clinical picture, MRI, CSF and blood biomarkers and nuclear medicine.

**Supplementary Information:**

The online version contains supplementary material available at 10.1007/s11547-022-01534-0.

## Key Results

1. The assessment of dementia is based on clinical, radiological, nuclear and lab evaluation.
2. Brain atrophy, white matter lesions, microhemorrhages, and vascular diseases should be radiologically evaluated.
3. Radiological assessment should be based on visual assessment, scoring, and volumetry.
4. The disease-oriented structured radiology report increases its clinical value.
5. Multidisciplinary teamwork increases diagnostic accuracy.

## Background

Dementias are taking up more space in everyday clinical and radiological scenarios, considering that the worldwide population is aging [78] and that brain imaging plays a key role in the assessment of cognitive impairment [8]. In addition, the scientific community is increasingly focused on the debate over the use of AI [51], FDA- and EC-approved automated segmentation software [73], and the optimal use of neuroimaging in new drug trials [68].” A recent survey, carried out in Europe among academic and non-academic institutions [74], disclosed that the current practice in dementia imaging presents some homogeneity (mainly in imaging acquisition and image interpretation) but also differences in training and reporting, in using advanced imaging techniques and volumetric measures, as well as in communication between clinicians and radiologists. This work stems from the need of different Italian scientific societies to standardize and optimize the radiological approach for the assessment and follow-up of the aging brain and cognitive disorders.

We aim to fill this gap of variability and uncertainty, providing a practical approach in evaluating, interpreting, and monitoring the aging brain and main cognitive disorders.

## Methods

The promoters of the initiative (FBP and SB) representatives of the Neuroradiological Section of the Italian Society of Radiology (SIRM), with the clinical support of EC and GBF, created a core panel with experts in dementia and cognitive disorders representatives of the Italian Association of Diagnostic and Interventional Neuroradiology (AINR). The purpose of their work is to submit a preliminary consensus draft to the representatives of SIGG (Italian Society of Geriatrics and Gerontology, Società Italiana di Geriatria e Gerontologia) and AIP (Italian Association of Psychogeriatrics, Associazione Italiana di Psicogeriatria) for their revision and final approval.

The main research questions were:

- Define the key concepts (A) of what the radiologist needs to know: main clinical features (definition of brain aging, cognitive syndromes, and primary and secondary dementias) and imaging findings.
- Frame the MRI radiological approach for the assessment and follow-up of the aging brain and cognitive disorders—MRI protocol (B), imaging evaluation and interpretation (C), and reporting (D) for clinical use
- Identify the main factors that will influence clinical and radiological practice in this population in the near future (E)
- The consensus between experts was reached using a similar approach to the previously published paper (Pizzini FB et. Insights Imaging) and consisted in:
  - A critical review of previous literature by European/American task forces and scientific societies related to A–D
  - Circulation and discussion of the draft based on this review among the core panel and then between the experts in more rounds
  - Changes of the original draft till the group converged towards an agreement on all the points A–E

### Literature review

**Fig. 1:**
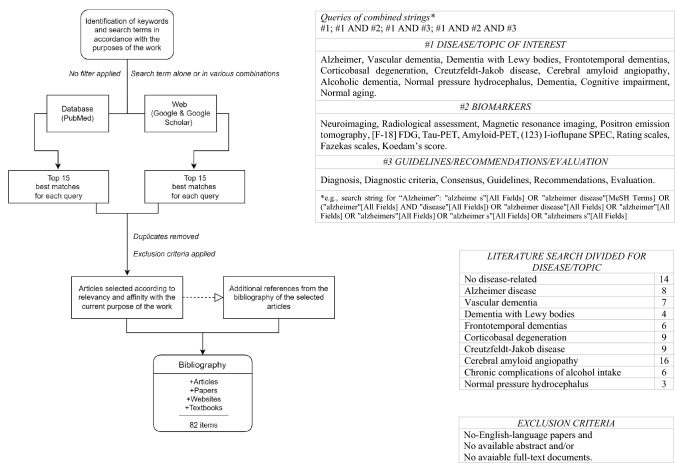
Flow-chart of bibliographic research and related tables

Literature review was performed through the PubMed database and on the web through Google and Google Scholar platforms, as well as specialized websites (Radiopaedia.org and radiologyassistant.nl/neuroradiology) and textbooks. The standardized strings used to search the database for literature were structured by combining the keywords (1) disease of interest, (2) biomarker (3) guidelines/recommendations/evaluation. As shown in the flowchart (Fig. 1), only original texts (abstract and full text) published in English were considered, without filtering for article type and publication date. Articles were selected after a review of the titles and abstracts of the first fifteen “best matches” to determine relevance and affinity to the research purpose. When it was useful, consultation was extended to the bibliographic references of the selected articles. Finally, in our bibliography, articles range from 1988 to 2022, with a predominance of the last decade.

## Results

**(A)** **Introduction to aging, cognitive impairment, and dementia**

The following boxes represent consensus findings related to the key concepts (Box 1) and clinical features and imaging findings (Boxes 2, 3, 4, 5, 6, 7, 8, 9, 10 and 11) of the major primary and secondary dementias.

**Box 1:** Glossary and definitions

**NORMAL AGING**: certain cognitive performances on formal testing are spared over the lifetime (e.g., visual perception/recognition), others decline after the sixth decade. These late-life changes involve especially the episodic and working memory, and executive function as well. ***Neuroimaging (NI) discloses variable structural brain findings, with an approximative rate of total brain volume loss per decade up to 5% after the age of 70***
**SUCCESSFUL AGING**: this term identifies older individuals who are free from chronic disease and continue to function well into old age (both physically and cognitively). ***NI reveals larger brain structure volumes with the absence of vascular lesions than the age-matched population***
**MILD COGNITIVE IMPAIRMENT (MCI):** intermediate clinical state between normal cognition and dementia (with increased risk for progressing). ***NI can detect signs of brain pathologies (atrophy, plaques, tangles, small and big vessels diseases, *etc**.)
**DEMENTIA**: decline in cognition involving one or more cognitive domains. Deficits represent a decline from previous level of function and interfere with daily function and independence. ***NI findings vary according to the etiology of the cognitive impairment***
**PRIMARY DEMENTIAS**: neurological diseases whose manifestations are predominantly cognitive. Most primary dementias are caused by neurodegenerative proteinopathies (e.g., tauopathy, prionopathies, α-synucleinopathies, etc.) which lead to neuronal loss and neuroinflammation with glial reaction. Neurodegenerative dementias include Alzheimer's disease (AD), Lewy body dementia (LBD), frontotemporal dementias (FTD), and prion diseases (sCJD). Vascular dementia (vaD), though not a neurodegenerative condition, is grouped together with primary dementias
**SECONDARY DEMENTIAS**: cognitive dysfunction is caused by ***structural lesions of the brain (e.g., normal pressure hydrocephalus [iNPH], brain tumors, *etc**.), nutritional deficiencies (vitamin B12 deficiency, folate deficiency, niacin, and thiamin deficiency), endocrine disease (hypothyroidism, hyperthyroidism, etc.), inflammatory disease (e.g., systemic lupus erythematosus, vasculitis, and sarcoidosis), infectious diseases (e.g., Whipple’s disease, Lyme disease, and AIDS), alcoholic dementia and metabolic causes (e.g., hypoxia and dialysis)
**REVERSIBLE DEMENTIAS**: secondary dementias are, to varying degrees, treatable and potentially reversible
**MIXED DEMENTIA:** also known as ***multiple etiology dementia***; it is a combination of multiple pathologies where each has a clinical impact (e.g. *vascular and degenerative, traumatic and vascular, *etc.)

See dedicated Boxes for further details about AD, FTD, CBD, sCJD, iNPH, CAA, Alcoholic dementias, DLB, VaD

**Box 2:** Late-onset Alzheimer Disease (Lo-AD)

Generals	– The most common cause of late-onset (≥ 65 yo) dementia [52] – Incidence and prevalence increase with age (growing up because of an aging population) [1] – Cerebrovascular disease frequently coexists with AD – Cerebral amyloid angiopathy (CAA) can co-occur with AD – General neurological examination is substantially normal – Neuropsychiatric symptoms are common		
Clinical features and diagnosis	Typical presentation: – Early and prominent episodic memory loss (recent events) *plus* – Executive, language, and visuospatial impairment	**MRI** – Disproportionate bilateral (or mild asymmetric) temporoparietal cortical atrophy with (early) prevalent involvement of entorhinal cortex/ medial temporal lobes (± hippocampus ┼) and (more later) precuneus ¶ and posterior cingulate gyrus – Relative sparing of the primary motor&somatosensory (pericentral) and occipital cortex **FDG-PET:** mirrors MRI findings, with gross correspondence between hypometabolic and atrophic areas *Δ* **Ioflupane-SPECT** (DaTscan): normal **Amyloid-PET**: abnormal diffuse cortex uptake with loss of gray-white differentiation (cerebellum spared) **τ-PET**: hippocampal-body and precuneus abnormal τ uptake in early-stage	
	Atypical presentation/non-amnestic syndromes (more common in early-onset disease [42]): – Visual variant—*posterior cortical atrophy* (PCA) – *Logopenic variant primary progressive aphasia* (lvPPA) – Progressive executive dysfunction		[*For clinical overview and imaging findings see *Box 3]
*Pitfalls:* – Iron-sensitive sequences should be performed to assess for hemorrhages associated with CAA (see Box 9) [22] * -* SVD *associated WMHs* frequent coexist with AD findings			

┼ Compared to early-onset AD, patients with late-onset disease show greater medial temporal atrophy and less cortical atrophy

^¶^ The involvement of precuneus is a late feature of lo-ad or an early feature of eo-AD [see Box 3]

*Δ* Usually the FDG-PET findings correspond to the MRI atrophic changes, but in some cases the molecular findings may be more severe than structural findings

**Box 3:** Early-onset Alzheimer Disease (Eo-AD: “not just an AD at a younger age”)

Generals	– The most common cause of early-onset (< 65 yo) neurodegenerative dementia [41] – AD with clinical onset younger than 65 yo – Better memory but worse attention/language/executive functions/visuospatial skills than Lo-AD	
Clinical features and diagnosis	Up to two-thirds of patients with non-amnestic phenotypic variants: – *Logopenic variant primary progressive aphasia ┼* lvPPA (most common): progressive decline in language (word-finding difficulty with hesitations in repetition) with relatively spared memory and cognition – *Posterior cortical atrophy*—PCA (second most common): progressive and disproportionate loss of visuospatial/visuoperceptual functions up to Gerstmann ¶ or Balint *Δ* syndrome – *Behavioral*/dysexecutive (aka *Frontal variant*): early apathy/abulia or disinhibition and impulsivity – *Acalculia variant* ◊ and other parietal syndromes (anomia, ideomotor apraxia, *Gerstmann* syndrome) – *Corticobasal syndrome* (CBS) [*see Box *7]	**MRI:** – Hippocampal sparing and less mesial temporal lobe disease than Lo-AD – Focal cortical atrophy – Greater posterior (parietal, temporoparietal junction, posterior cingulate cortex) neocortical atrophy than Lo-AD – Parieto-occipital and posterior temporal lobe involvement (PCA) – Left posterior temporal cortex and the inferior parietal lobule involvement (lvPPA) – Involvement of bilateral temporoparietal regions along with milder involvement in the frontal cortex (frontal variant) – Biparietal involvement, greater on the left (parietal syndromes) **FDG-PET:** mirrors MRI findings, with gross correspondence between hypometabolic and atrophic areas **Ioflupane-SPECT** (DaTscan): normal **Amyloid-PET:** as in Lo-AD [see Box 2] **τ-PET:** – Relatively distinct areas of uptake (tau uptake correlates with clinical symptoms [6, 79]) – Possible more uptake than Late-onset-AD due to increased Tau burden in posterior neocortices
*Pitfall:* if suspicious of PCA is clinically high, the absence of marked parieto-occipital atrophy should not exclude the diagnosis		

┼ Other forms of PPA, such as the nonfluent/agrammatic and semantic variants, are non-Alzheimer syndromes due to FTLD [see Box 6]

^¶^ Gerstmann syndrome: acalculia, left–right disorientation, finger agnosia, and agraphia

*Δ* Balint syndrome: ocular motor apraxia, optic ataxia, and simultanagnosia

◊ These patients overlap with or may eventually meet current criteria for posterior cortical atrophy or corticobasal syndrome

**Box 4:** Vascular dementia (VaD)—Vascular cognitive impairment (VCI) “not strictly a neurodegenerative dementia ┼”

Generals	– The second most common type of dementia (after AD) – May occur with single or multiple infarcts, cortical or subcortical – “Silent” strokes are significant risk factors – Often *multiple-etiology dementia* (VaD *plus* AD) – “Classically” stepwise dementia
Clinical features & diagnosis	Two main VaD syndromes: – *Poststroke dementia (common)*: stepwise cognitive decline *plus* clinically diagnosed stroke – *Vascular dementia without recent stroke* (aka *Binswanger* disease ¶ and *subcortical ischemic vascular dementia*): progressive or stepwise cognitive decline *plus* brain neuroimaging evidence of cerebrovascular disease
	Main stroke signs & symptoms (according to arterial vascular territory): – Anterior cerebral artery → motor and/or sensory deficit, gait apraxia, abulia *Δ* – Middle cerebral artery: ∙ Dominant hemisphere: aphasia, motor and sensory deficit up to hemiplegia, and hemianopia ∙ Non-dominant hemisphere: motor and sensory deficit, homonymous hemianopia, neglect – Posterior cerebral artery → hemianopia, alexia without agraphia, visual hallucinations, sensory loss, conjugate gaze palsies, motor deficit – Penetrating vessels (lacunar syndromes) → pure motor hemiparesis, pure sensory deficit, pure sensory-motor deficit, ataxic hemiparesis, dysarthria/clumsy hand – Vertebrobasilar ◊ → cranial nerve palsies, crossed brainstem syndrome, diplopia, dysarthria, dysphagia, dizziness, nausea, vomiting, ataxia, coma – Internal carotid artery → MCA symptoms usually preceded by contralateral amaurosis fugax
	**MRI:** variable appearance and location (usually multifocal and asymmetric abnormalities) as follows § [14, 22, 56, 58]: ∙ Large vessel or atherothromboembolic disease – Multiple infarcts (e.g., bilateral anterior cerebral artery infarction, and parietotemporal *plus* temporo-occipital infarction of the dominant hemisphere) – Single strategically placed infarct → medial thalamus; lateral thalamus—internal capsule; caudate and pallidus; posterior cerebral artery infarction (with infarction of the paramedian thalamic region and inferior medial temporal lobe of the dominant hemisphere); left angular gyrus (Gerstman’s syndrome; basal forebrain infarction) ∙ Small vessel disease (relevant role) – Multiple (> 2) lacunar infarcts in (frontal) white matter and deep gray matter nuclei ¥ – Ischemic white matter changes (WMLs) involving at least more than 25% of the whole WM – Dilatation of perivascular spaces – Cortical microinfarcts and microhemorrhages ∙ Hemorrhage: – Intracerebral hemorrhage – Multiple cortical and subcortical microbleeds – Subarachnoid hemorrhage ∙ Hypoperfusion – *Hippocampal sclerosis* – *Laminar cortical sclerosis* – Watershed infarcts (*aka* border zone infarcts) in the dominant hemisphere (superior frontal and parietal regions) ╪ **FDG-PET:** mirrors MRI findings, with gross correspondence between hypometabolic and atrophic areas **Ioflupane-SPECT (DaTscan):** normal uptake **Amyloid-PET:** not useful (maybe abnormal uptake) **τ-PET:** no uptake
*Pitfall:* – WMH burden, even if moderately severe, is not sufficient evidence for VaD (WMH are not specific for cerebrovascular disease) – AD MRI findings may coexist – Besides “classical” types of vascular lesions (i.e., atherosclerosis, cardiac/atherosclerotic/systemic emboli, cerebral venous thrombosis, arteriolosclerosis, etc.) consider angiopathies (with and without inflammation), arteriovenous fistulas, hereditary angiopathies (e.g., CADASIL, CARASIL, etc.) and *CAA *[see Box 9] – In case of acute onset of cognitive impairment (including *delirium*) and/or focal neurological signs/symptoms (hemispheric and/or vertebrobasilar), perform urgent brain CT and CT angiography ± advanced neuroimaging (i.e. perfusion/DWI studies) to assess acute stroke/stroke mimics and guide treatment (e.g., IVT and/or EMT if ischemic stroke)	

┼ Vascular-induced dementia should be included in the differential diagnosis for patients with neurocognitive impairment. however, VaD should be considered a secondary dementia, not a neurodegenerative disorder [see Box 1]

¶ The term Binswanger disease is currently out of use

Δ Also account for anterior cranial fossa lesions and NPH in the differential diagnosis [see Box 11]

◊ Notably, bilateral findings indicate basilar artery involvement up to the “top of the basilar” syndrome

^§^ Adapted from VASCOG work, p. Sachdev et al*. 2014*

¥ Notably, multi-infarct dementia represents a subtype of VaD (not a synonymous as in the past)

╪ Watershed cerebral infarctions are due to hypoperfusion or microemboli. Two types can be distinguished, with corresponding pathological/radiological aspects:

– Cortical → cortical laminar necrosis (early cytotoxic edema [↑DWI/↓ADC], T1 curvilinear hyperintensities, ↑/ ~ *T2/FLAIR)*

Deep → pearl thread sign (DWI and T2/FLAIR signal abnormality as a series of rounded areas adjacent to the lateral ventricle) [57]

**Box 5:** Dementia(s) with Lewy Bodies—DLB (Lewy Body Dementias – LBDs)

Generals	– The third most common cause of late-onset dementia (after AD and vascular dementia) – Lewy Body Dementia as “umbrella term”: *clinical* diagnoses of both *Parkinson disease dementia (PDD)* and *dementia with Lewy* bodies (≠ *Lewy body disease:* pathologic diagnosis)
Clinical features & diagnosis	– Core clinical features (first three occur early): 1. Fluctuating cognition with variations in attention and alertness 2. Visual hallucinations 3. *REM sleep behavior disorder* 4. One or more spontaneous cardinal features of parkinsonism (bradykinesia, rest tremor, rigidity, postural instability) – Other common symptoms: autonomic dysfunction, antipsychotic drug sensitivity, repeated falls – Visual processing, attention, and executive functioning more compromised than memory and naming
	**MRI** (bare and non-specific) – (Mild) cortical atrophy in the occipital lobes, with sparing of the posterior cingulate gyrus (*cingulate island sign* ┼) – Relative preservation of the mesial temporal lobe and hippocampus – Absent “swallow tail sign” on axial high-spatial-resolution SWI [64] **FDG PET**: mirrors MRI findings, with gross correspondence between hypometabolic and atrophic areas **Ioflupane-SPECT (DaTscan)**: *bilateral* (sometimes asymmetric) loss of uptake beginning in the putamen and later spreading to the caudate head **Amyloid-PET and τ PET**: not useful

┼ The cingulate island sign is useful in differentiating DLB from AD, in which the posterior cingulate gyrus is usually involved [21]

**Box 6:** Frontotemporal dementias (FTDs)

Generals	– The fourth most common cause of late-onset dementia; second-one of early-onset dementia (after Eo-AD) [30] – *Frontotemporal lobar degeneration* (FTLD, in which *Pick-disease is included*) denotes the pathological diagnoses associated with clinical FTDs	
Clinical features & diagnosis	– All FTD-forms show behavioral disturbances and whole cognitive function impairment – Clinical presentation (first two are the main groups): *∙ Behavioral variant FTD* (bvFTD, most common): progressive personality and behavior changes. Up to 20% of patients develop *motor neuron disease* (MND) ┼[32, 63] *∙* Primary *progressive aphasia* (PPA), two variants (of three total canonical variants) ¶[20]: 1. Nonfluent/agrammatic *variant PPA* (nfvPPA): effortful and interrupted speech 2. *Semantic variant PPA* (svPPA): impaired comprehension and naming with preserved fluency, repetition, and grammar * ∙* Motor syndromes with FTD: MND, *corticobasal syndrome* (CBS), and *progressive supranuclear palsy* (PSP) – “Probable bvFTD” requires a supportive MRI scan (while “possible bvFTD” on clinical features basis alone)	**MRI:** – Bilateral frontal and temporal involvement with anterior to posterior gradient (if severe, knifelike gyri) * ∙* Predominant changes in the frontal and temporal lobes, with asymmetric involvement of the anterior temporal lobes, prefrontal cortices, insula, anterior cingulate, striatum, and thalamus [61] (bvFTD) * ∙* Left more than right anterior perisylvian involvement (lvPPA-nfvPPA) * ∙* Left (70%) more than right involvement of temporal cortex & parietal lobe (lvFTD-svPPA) * ∙* Focal anterior temporal pole involvement (svPPA) – (Asymmetrical) hippocampal atrophy, more pronounced anteriorly – Disproportionate widening of the frontal horns – Relative sparing of precuneus and occipital lobes *Δ* **FDG PET:** mirrors MRI findings, with gross correspondence between hypometabolic and atrophic areas **Ioflupane-SPECT (DaTscan)**: normal **Amyloid-PET**: not useful **τ-PET**: unclear role (variable uptake depending on variants)
		Structural MRI findings predict pathology ◊: – Severe “knife-edge” frontal and insular atrophy → Pick disease – Midbrain atrophy → PSP Focal thalamic atrophy → C9orf72 expansion – Ventral frontal pattern *plus* paper-thin anterior caudate nuclei → FTLD-FUS – Severe dorsal frontoparietal atrophy → concern for AD
Pitfall: patients with bvFTD may occasionally have a normal MRI		

┼ BvFTD, as well as the nfvPPA, also occur in the late stage of CBS and PSP [53]

^¶^ The logopenic variant is typically associated with Alzheimer pathology

*Δ* The relative sparing of precuneus and occipital lobes is useful in differentiating FTD from ad and DLB

◊ Three pathological subtypes (based on the composition of the protein inclusions) are recognized:

1. FTLD-tau (most common): several conditions, including pick disease, chronic traumatic encephalopathy, CBD, PSP

2. FTLD-TDP: includes the c9orf72 repeat expansion form (most common genetic cause)

FTLD-fet (less common, up to 10% of cases): fus gene involvement (pathogenic variants linked to familial ALS)

**Box 7:** Corticobasal degeneration (CBD)

Generals	– Rare (precise incidence and prevalence unknown) atypical parkinsonism – *Corticobasal syndrome* (CBS) for clinical diagnosis, CBD if neuropathologic confirmation – Challenge clinical diagnosis (wide variety of presentations)
Clinical features & diagnosis	Signs and Symptoms (characteristic but not specific): – Behavioral and/or cognitive [43] disturbance → executive dysfunction, aphasia (variable entity, up to *nonfluent variant PPA*), apraxia (various types), visuospatial dysfunction – Motor symptoms → progressive asymmetric (sometimes symmetric [29]) movement disorder initially affects one limb (upper or lower; then progression to all four limbs) with various combinations of akinesia, rigidity, dystonia, focal myoclonus, ideomotor apraxia, *alien-limb phenomena* (up to 50% of patients) and gait disturbance (various types)
	**MRI**: – Normal in early stages – Asymmetric (contralateral to the more clinical affected side) and severe focal atrophy of the posterior frontal and parietal regions, along with dilatation of the lateral ventricles (in up to half of the patients) – Atrophy of paracentral cortex – Atrophy of the corpus callosum [80] – T2-hyperintensity of the atrophic cortex and underlying white matter ± hypointensity of the putamen and pallidus [59, 67] Atrophy of the frontal lobes, basal ganglia, and brainstem on MRI Voxel-based morphometry [35] **FDG-PET**: – Asymmetric hypometabolism in the posterior frontal (premotor and supplemental motor), inferior parietal, and superior temporal regions, thalamus, and striatum of the more affected hemisphere [16] – Asymmetric decrease of global cortical oxygen consumption [44] **Ioflupane-SPECT (DaTscan):** asymmetric reduced uptake (uniform reduction throughout the striatum ≠ greater putaminal loss in PD) **Amyloid-PET**: not useful **τ-PET** ┼: not useful
*Pitfall*: focal atrophy detected on MRI by voxel-based assessment in premotor and supplemental motor areas is suggestive of CBD or PSP, while more widespread atrophy is suggestive of FTLD or AD	

┼ To date, there are not available tau-PET ligands for detecting CBD straight filament 4repeat tau disease in clinical routine [70, 81]

**Box 8:** Sporadic Creutzfeldt-Jakob disease (sCJD) ┼

Generals	– Rare disease (incidence 1/1.000.000; mean age 62yo) – Most frequent (90%) of sporadic human prion disease
Clinical features & diagnosis	– Two cardinal clinical manifestations: ∙ Rapidly progressive mental deterioration (akinetic mutism in the end-stage) ∙ Myoclonus (90%) – Gait disturbance and various among pyramidal (especially in end-stages), extrapyramidal, and cerebellar manifestations (presenting symptoms in up to 40% of cases) – Brain MRI is the most sensitive diagnostic test in the early stages
	**MRI**: – FLAIR/T2-hyperintensity and DWI reduced diffusivity (unilateral or bilateral; focal, multifocal, or diffuse; and asymmetrical or symmetrical in early stages, then tendency to greater symmetrical involvement) in the head of the caudate and putamen (lesser extent in the thalamus) – Cortical ribbon especially involving the superior frontal gyrus, superior parietal lobule, cingulate gyrus, and insular cortex – Perirolandic cortex is usually spared – Generalized atrophy and ventricular dilatation (prominent in later stages) – Rare isolated limbic involvement – Possible late Cerebellar atrophy ¶ – Confluent hyperintense signal in the mesial and dorsal thalami on DWI, FLAIR, and T2-weighted MRI ("double hockey stick" or "pulvinar" sign) → typical of variant CJD (vCJD or 'mad cow disease’, now rare) and rare cases of sCJD MRI abnormalities may vary with the clinical syndrome and molecular subtype [40] *Δ*: – Increased T2 signal in the caudate and putamen → early dementia, shorter survival, and VV2, MV2, or MM1 codon 129 genotypes – Thalamic hyperintensities → VV2 and MV2 subtypes – Increased wide cortical signal → VV1 and MV1 subtypes – Normal MRI or only late atrophy or white matter changes → MM2 thalamic form [27] **PET-FDG**: not sufficiently evaluated **Ioflupane-SPECT (DaTscan**): possible asymmetric loss of uptake [10] **Amyloid-PET**: crossreaction with prion proteins reported [39] **τ-PET**: normal [13]
*Pitfalls*: – DWI is the most sensitive MRI sequence for the detection of CJD-related cortical and striatal changes [38] – MRI-findings are not fully specific for CJD (consider stroke, vasculitis, or reversible posterior leukoencephalopathy)[5, 66]	

┼ Three forms of Creutzfeldt-Jakob disease are recognized:

– Sporadic (sCJD, 85–95%)

– Genetic (gCJD, 5–15%)

– Acquired: iatrogenic Creutzfeldt-Jakob disease (iCJD, < 1%), and variant Creutzfeldt-Jakob disease (vCJD, < 1%)

^¶^ Although the prevalence of cerebellar symptoms and neuropathological findings, DWI- and FLAIR-hyperintense signal in the cerebellum is not typical [12]

*Δ* Subtypes of sCJD are classified according to the genotype of the prion protein (prnp) gene codon 129 and the molecular properties. Clinical phenotypes agree with molecular subtypes (e.g., **MM1 and MV1** correlate with the "classic CJD"; **VV2** is the “ataxic variant”, **MM2** can present as either a thalamic variant or a cortical variant, etc.)

**Box 9:** Cerebral amyloid angiopathy (CAA)

Generals	– Frequent cause of intracerebral hemorrhage (ICH) and cognitive impairment in the elderly – Age-dependent incidence (rare < 60 yo) – No clinical overlap between CAA and non-central nervous system systemic amyloidosis			
Clinical features & diagnosis	***Acute lobar intracerebral hemorrhage*** (ICH, commonly older adults)	**MRI**: – Commonly in posterior (temporal and occipital) lobar brain regions – Possible cerebellum involvement (with a predilection for cortex and vermis) – Possible extension into the subarachnoid (± “finger-like” projections [55]), subdural spaces, and into ventricles (less frequent) – Leptomeningeal vessels deposition: (1) Potential source for isolated convexity subarachnoid hemorrhage (cSAH) (2) cortical superficial siderosis (cSS, in chronic phase along with cortical microbleeds [CMBs] on T2*sequences)		
	***Transient focal neurologic episodes ***(TFNE, aka “amyloid spells”): recurrent, brief and stereotyped spells of cortical symptoms (e.g., weakness, numbness, paresthesia, etc.)		**MRI** with gradient echo or other T2*-weighted sequences identifies cSAH, cSS, or cortical microbleeds (CMBs) in the region of cortex corresponding to TFNE symptoms	
	***Cerebral amyloid angiopathy-related inflammation*** (CAA-ri, inflammatory response to amyloid deposition): – Acute/subacute/progressive cognitive decline (~ 75%), headache (~ 40%), ↓ consciousness, behavioral change, or focal neurological signs (e.g., seizures ~ 30%) – Younger than those with others CAA-related manifestations		**MRI** (with *contrast* ┼): – T2/FLAIR patchy or confluent immediately subcortical white matter hyperintensities (leukoencephalopathy) in subcortical white matter (often asymmetric) + multiple lobar microhemorrhages (microbleeds) on T2* sequences – Gadolinium enhancement in ~ one-third of cases -CAA-ri [2] – No evidence of large– or medium-vessel vasculitis on (MRA or angiography)	
	***Cognitive impairment*** (coexisting AD and/or VCI)		**MRI**: variable overlap of typical imaging-findings of CAA, VCI and AD	
	**Incidental imaging findings** → Chronic evidence of asymptomatic bleeding detected on brain MRI of patients with or without lobar hemorrhage Include: – (Cortical) microbleeds (microhemorrhages, up to about 25% elderly population): 2–10 mm focal areas of hemosiderin deposition on T2*-sequences (predilection for the cerebral cortex) – Cortical superficial siderosis ¶ (cSS, maybe the chronic form of acute convexity subarachnoid hemorrhage related to CAA; up to 60% of CAA):			
	**MRI hemorrhagic findings** (imaging-based diagnosis of CAA): ** ∙ Modified Boston criteria** [36]**:** combination of clinical, radiographic and pathological criteria. Four tiers: (i) Definite (post-mortem) (ii) Probable with supporting pathological evidence (iii) Probable CAA, with MRI: – Multiple hemorrhages restricted to lobar, cortical, or cortical-subcortical regions (cerebellar hemorrhages allowed) without another cause, **or** – Single lobar, cortical, or cortical-subcortical hemorrhage **and** focal (three or fewer sulci) or disseminated (more than three sulci) cortical superficial siderosis without another cause (iv) Possible CAA, with MRI: – A single lobar, cortical, or cortical-subcortical hemorrhage without another cause, **or** – Focal or disseminated cortical superficial siderosis without another cause ∙ CAA-ri → symptomatic patients with diagnostic imaging evidence of inflammation and CAA-related hemorrhagic findings *plus* exclusion of other causes [11] **Amyloid-PET** (using early-phase 11C-PiB PET *Δ*) and (resting-state [18F])**FDG-PET:** – Lower occipital/posterior cingulate (O/PC) tracer uptake ratio in probable CAA than AD – Regions with high PiB uptake area associated with subsequent hemorrhage [26] **τ-PET**: not useful			**MRI non-hemorrhagic findings**: – Acute ischemic microinfarcts: asymptomatic punctate hyperintense lesions on DWI[71] – Cerebral atrophy (most pronounced in occipital regions and more severe in higher cortical microbleeds burden – WMH (T2/FLAIR): frequently small-vessel disease – Centrum semiovale dilated perivascular spaces ◊
*Pitfalls*: – Patients with isolated incidental hemorrhagic imaging findings (i.e., a single CMB or equivocal cSS) should undergo to follow-up to detect the development of progressive subclinical findings to further support the diagnosis of CAA (sec. modified Boston Criteria) – Consider lobar extension of a hypertensive hemorrhage, hemorrhagic transformation of an ischemic stroke, hemorrhagic venous infarction from cerebral venous thrombosis, hemorrhage of AVM, and hemorrhagic tumor as differentials of nontraumatic lobar ICH – Microbleeds commonly arise from either CAA or hypertensive vasculopathy (small-vessel disease) § – Mind the possible development of CAA-ri-like findings (aka “Amyloid Related Imaging Abnormalities”, ARIA) in patients treated with anti-beta-amyloid immunotherapy for AD ¥ [3, 68] – AD, VCI, and CAA MRI findings may coexist				

┼ Notably, MRI with contrast should be performed in only a few cases in the assessment of dementia, in particular, contrast-enhanced MRI should be performed only in a few cases in the evaluation of dementia, especially in subacute/acute and rapidly progressive cases

^¶^ If disseminated, cSS indicates a higher risk for future ICH. Notably, neither cSS nor superficial siderosis involving the cerebellum or brainstem is specific of CCA (possible comparison in, e.g., reversible cerebral vasoconstriction syndrome, primary angiitis of the CNS, bacterial endocarditis, hyperperfusion syndrome after carotid endarterectomy, cerebral venous sinus thrombosis, posterior reversible encephalopathy syndrome, dural tear and craniospinal surgery, etc.) [9, 33, 37]

*Δ* The early-phase 11c-PiB PET help differentiate probable CAA from probable AD. Early-phase amyloid PET images are used as a surrogate for brain perfusion, as opposed to the standard late-phase 11c-PiB PET/CT which reflects binding to aβ deposits

◊ When predominantly placed in the centrum semiovale, the dilated perivascular spaces are frequently associated with CAA [72]

^§^ Microbleeds are not specific for CAA as they can be seen in multiple conditions, including, hypertension, cerebral cavernous malformations, coagulopathy, thrombocytopenia, anticoagulant medications, CNS vasculitis, infective endocarditis, end-stage kidney failure. Moreover, beside conditions above-mentioned, microbleeds are also more prevalent among users of antiplatelet agents [24]. Notably, while microbleeds (in general) are not specific of CAA, cortical ones (i.e., those limited to the cerebral and/cerebellar cortex and vermis), suggest CAA [48] (although not pathognomonic). In contrast, deep microbleeds (those involving the basal ganglia, thalamus, or pons) are presumably of hypertensive microangiopathic origin

¥ In those with ad treated with immunotherapy, CAA may develop because of the rapid destruction of parenchymal aβ, and/or ARIA may be triggered if CAA is preexistent to the immunotherapy

**Box 10:** Chronic central nervous system complications of alcohol (ab)use [overview of main conditions]

Clinical features and diagnosis	***Korsakoff syndrome*** (“a residual syndrome”) ┼: – Memory disorder with selective anterograde and retrograde amnesia (which lead to confabulation) with apathy and relative preservation of long-term memory and other cognitive functions Most frequently represents a late neuropsychiatric manifestation of WE in alcohol abusers (80% of WE episodes), but also caused by a “non-alcoholic” deficit of thiamine – Sometimes occurs without a clinical recognized WE (that may have been subclinical or unrecognized) – No *clear correlation between neuroimaging and severity/duration of alcohol abuse or degree of cognitive impairment* – Rare recovery (abstinence and nutritional repletion)	**MRI**: – Lesions in the anterior thalamus (→ correlate with memory impairment) [28] Areas of disproportionate focal volume loss/atrophy (relatively specific)[50]: 1. Mamillary bodies (a specific sign of prior WE) 2. Medial thalamus 3. Corpus callosum
	***Cognitive impairment***: – 50 to 70% of chronic alcohol abusers on neuropsychological testing – Role of ethanol neurotoxicity – No *unequivocal evidence of brain lesions which are caused solely by chronic ethanol ingestion* – Selective loss of neurons in frontal regions (mirrored on FDG-PET) – Possible recovery with abstinence	**MRI/TC**: – Ventricular and sulcal enlargement that does not correlate with the severity ¶ – Disproportionate diffuse ↓ of subcortical white matter compared with cortical gray matter [50] *Δ* FDG-PET: frontal regional hypometabolism
	***Alcoholic cerebellar degeneration:*** – Chronic cerebellar syndrome – After 10 or more years of excessive alcohol use – Slow development (over weeks to months or years) of abnormal stance, gait, and lower extremity coordination (sometimes abrupt onset and/or worsening – Cognitive function is usually spared (except in cases of prior WE) – Abstinence may prevent worsening	**MRI/TC**: – Rule out mass lesions or others – Cerebellar cortical atrophy – Prominent atrophy of the anterior vermis (help distinguish from degenerative conditions with diffuse atrophy) FDG-PET: a research tool
	***Marchiafava-Bignami disease:*** – Rare disorder (probable undiagnosed) – Demyelination or necrosis of the corpus callosum and adjacent subcortical white matter – Malnourished alcoholics – Dementia, spasticity, dysarthria, and inability to walk (acute, subacute, or chronic course) – Variable prognosis (coma and death, survival in a demented condition, interhemispheric disconnection syndrome, occasional recovery)	**MRI/CT**: – Hypodense lesions in the corpus callosum (CT) – Discrete or confluent areas of decreased T1 signal and increased T2 signal in the corpus callosum (MRI)
*Pitfall*: Knowledge of typical and atypical imaging findings of WE, particularly when the clinical presentation is non-specific, is mandatory since timely administration of thiamine may halt brain damage, thus preventing the development of Korsakoff Syndrome		

┼ The Korsakoff syndrome is part of the Wernicke-Korsakoff syndrome (WK)—the best-known neurologic complication of thiamine (vitamin b1) deficiency—which encompasses two different syndromes:

(1) the acute Wernicke encephalopathy (WE) syndrome

(2) the chronic Korsakoff syndrome (KS, usually a consequence of WE)

There is no generally accepted definition of KS, nor generally accepted criteria for the diagnosis of KS

^¶^ Several studies showed that the ventricles and sulci become significantly smaller with short-term alcohol abstinence (approximately one month of abstinence). Notably, cognitive abnormalities improve too with alcohol abstinence [4, 62, 82]

*Δ* Similar to the reduction in ventricular dilation achieved with short-term abstinence, the amount of white matter also increases in response to alcohol withdrawal, suggesting a reversible damage [19]

**Box 11:** Idiopathic Normal Pressure Hydrocephalus—iNPH ┼

Generals	– Insidious type of communicating hydrocephalus without substantially increasing CSF pressure – Rare and treatable disease (“reversible dementia”) – A correct diagnosis of NPH allows the selection of patients who will respond to shunt surgery			
Clinical features & diagnosis	Clinical features (neither pathognomonic nor specific): – Usually gait abnormality; uncommon full Hakim & Adams triad (gait abnormality, incontinence, and cognitive impairment with frontal syndrome) – Bradykinesia (55% of cases)			
	**MRI** → symptomatic patients (*features of iNPH*): – Ventriculomegaly (fourth spared; Evans’s index > 0.3 ¶) – Steepening of the callosal angle (CA < 90°, indirectly expresses DESH) *Δ* – Ventriculomegaly out of proportion to generalized sulcal enlargement – Narrow sulci and subarachnoid spaces at the vertex and medial/parafalcine region – Recognize DESH (specific of iNPH, ↑positive but ↓negative predictive values) ◊ – Look for congenital factors (e.g., aqueductal stenosis or webbing) → secondary NPH §			**MRI** → asymptomatic patient (incidental iNPH features): – Asymptomatic ventriculomegaly with *features of iNPH* on MRI (**AVIM**)
	**FDG-PET, DAT-scan, τ-PET**: help detect concomitant degenerative disease; do not have established findings suggestive of NPH **SPECT** (reflects the morphological changes of DESH) → convexity apparent hyperperfusion (CAPPAH) sign (specific of iNPH): – Decreased cerebral blood flow (CBF) in the anterior parts of the cerebral hemisphere and Sylvian fissure periphery – Relatively increased CBF in high cortical areas			
	Diagnostic criteria (probable iNPH)[45]	1. Meets the criteria for *possible iNPH ¥*		
		2. CSF pressure of 200 mmH2O or less and normal CSF content		
		3. One of the following two investigational features:	(a) Neuroimaging features of narrowing of the sulci and subarachnoid space over the high-convexity/midline surface (DESH) with gait disturbance (small stride, shuffle, instability during walking, and increase in instability on turning)	
			(b) Improvement of symptoms after CSF tap test and/or drainage test	
*Pitfall*: Incidental iNPH features configuring AVIM are an indication for further clinical evaluations and follow-up				

┼ NPH syndrome can be primary (idiopathic NPH), or secondary to some condition that impairs CSF absorption (e.g., following infectious, inflammatory, or hemorrhagic events involving the subarachnoid space)

^¶^Evans’s index is the ratio of the largest width of the frontal horns and the widest measure of the inner table of the skull at that level. When this ratio is greater than 0.3, the ventricles are considered enlarged [54]

*Δ* The CA helps to predict the effect of shunt intervention and is useful in differentiating iNPH from AD with a sensitivity of 97%, a specificity of 88%, and a positive predictive value of 93% at a cutoff value of 90° [31]. Notably, compared to AD, atrophy of the hippocampus is mild in iNPH

◊ The term DESH (included in the Japanese guideline for the diagnosis and treatment of NPH) describes prognostic MRI features in NPH, including a “tight high convexity” and enlargement of CSF spaces in the Sylvian fissure. DESH correlates with a good response to shunting

^§^Notably, symptoms due to stenosis of the cerebral aqueduct may manifest in adulthood, in form of syndrome long-standing overt ventriculomegaly in adults (LOVA), which has a clinical presentation like NPH. Secondary NPH should be suspected in a patient with large head size and MRI disclosure of triventriculomegaly without the involvement of the fourth ventricle, mild or no T2/FLAIR signal change around the ventricular system, and evidence of aqueductal stenosis and/or webbing identified with sagittal fast imaging employing steady-state acquisition c (fiesta-c) sequences

¥ Possible iNPH is diagnosed if the following criteria are met:

1. More than one symptom in the clinical triad: gait disturbance, cognitive impairment, and urinary incontinence

2. Above-mentioned clinical symptoms cannot be completely explained by other neurological or non-neurological diseases

3. Preceding diseases possibly causing ventricular dilation (including subarachnoid hemorrhage, meningitis, head injury, congenital/developmental hydrocephalus, and aqueductal stenosis) are not obvious. Instead, only the “shunt responder” can be meet the diagnosis of definite iNPH [45]

**(B) MRI protocol (acquisition**)

1. *Field of the MRI suite (1.5; 3T).* The choice of field strength does not affect the evaluation of atrophy or white matter load (WML), but it can affect the detection rate of microbleeds [46].
2. *Standard protocol (core sequences and parameters).* They have been found to have a major impact on image resolution [15] thus on the detection rate of atrophy, white matter changes, and microbleeds (e.g. in GRE detection of 33% of the microbleeds identified by thin-section SW [46, 65]) (Table 1).
3. *Optional additional sequences* (i.e. functional MRI, microstructural DWI, spectroscopy, Magnetization Transfer Imaging) are useful at the group level [25] when comparing a specific disease with healthy subjects or other clinically overlapping diseases, Level 1 of a five-level scale of Imaging biomarkers [18, 77]. While ASL (Arterial Spin Labeling) is useful at the individual level because it reaches a sensitivity and specificity > 80% for the clinical diagnosis of a given patient (Level 2 of the same scale). None of these sequences can be considered effective for early clinical diagnosis (Level 3) or could be used as surrogate criteria for pathological diagnosis (Level 4) or provide a direct measure of the underlying neuropathological changes (Level 5).
4. *Contrast-enhanced MR*: Contrast Media injection not indicated in aging and cognitive impairment (except for CAA-ri, see Box 8. CAA).

**Table 1:** MRI protocol

Sequence	Acquisition modality	Slice thickness/voxel size	Findings
T1-weighted	Three dimensions (3D), in axial or sagittal plane	< 1 mm isotropic	Grey matter atrophy Coronal plane: hippocampal atrophy Sagittal/axial plane: cortical atrophy
T2-FLAIR	Two-dimensions 2D axial/3D in sagittal plane	Minimum 1 mm for 3D, Minimum 3 mm for 2D	Atrophy, white and gray matter signal abnormalities Coronal plane: hippocampal signal abnormalities
TSE/FSE T2-weighted	2D axial	Minimum 3 mm	White and gray matter signal abnormalities, particularly thalamus and posterior fossa
Diffusion-weighted imaging	2D axial	Minimum 3 mm for 2D	Recent ischemic lesions
T2*-weighted/SWI	Two-dimensional 2D axial/3D in sagittal plane	Minimum 1 mm for 3D, Minimum 3 mm for 2D	Microbleeds, superficial hemosiderosis, hemorrages

**(C)** **Evaluation and interpretation**

1. Visual (qualitative) assessment

Table 2.

**Fig. 2:**
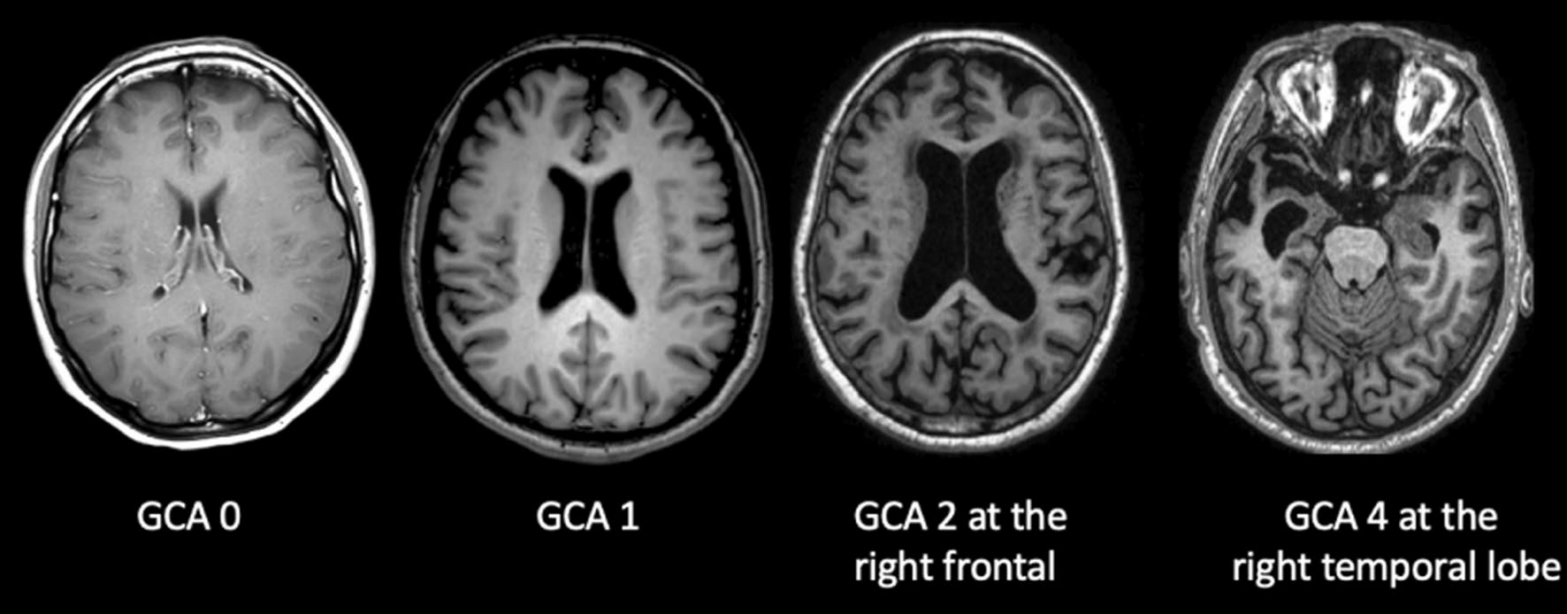
Axial T1 weighted images showing with increasing GCA score values from left (GCA = 0) to right (GCA = 3). The score reported is referred to the most affected brain area

**Fig. 3:**
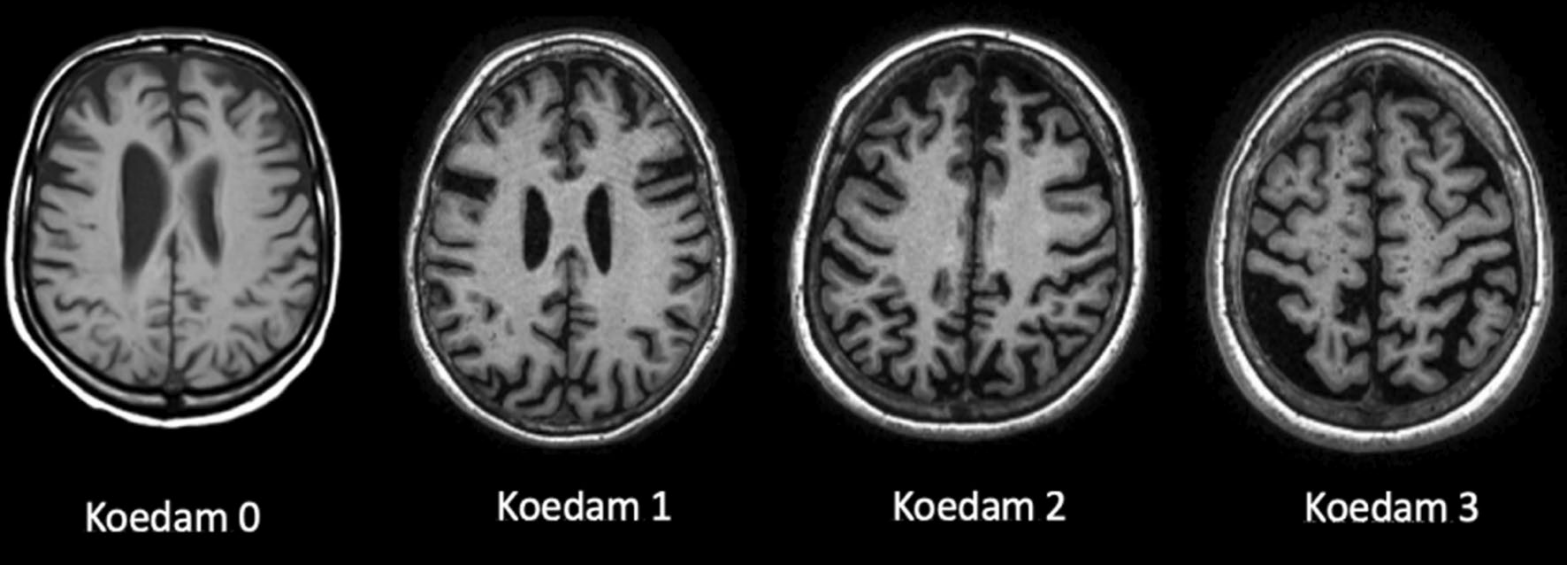
Axial T1 weighted images showing increasing parietal atrophy from left (Koedam = 0) to right (Koedam = 3)

**Fig. 4:**
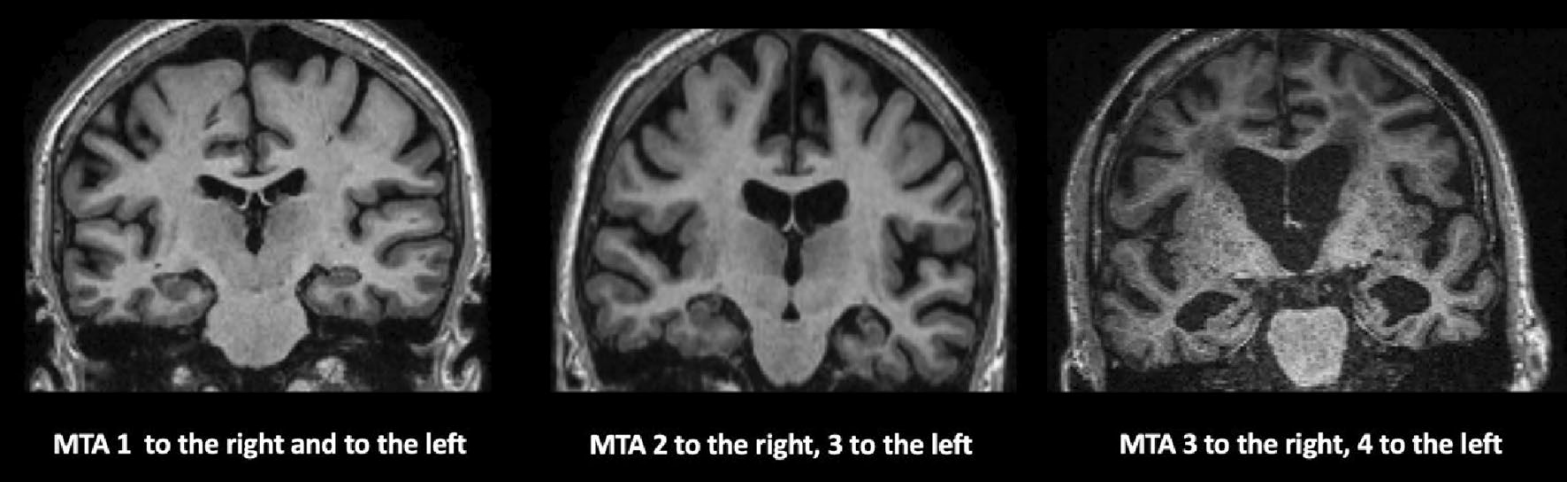
T1 weighted coronal images placed symmetrically and perpendicularly to the long axis of the hippocampi. Different grades of medial temporal lobe atrophy are shown, and they are rated both on the left and on the right hippocampi

- Visual rating *scales*
- ATROPHY (see Tables 3 and 4; Figs. 2 and 3, and 4) should be evaluated in multiple planes on T1 (or FLAIR, but not T2), comparing the most preserved sulci/gyri (usually the occipital ones) to the most affected ones, symmetrically, using these scales:

**Table 2:** Qualitative visual assessment

What to check	How to report	What to report
*Atrophy* (*in T*1)		
Ventricles	Evaluate size, symmetry, topography, associated findings	Ventriculomegaly and also disproportionate sulcal enlargement
Perivascular spaces	Evaluate size, symmetry, topography, associated findings	Severe and diffuse enlargement (> 3 mm) associated to atrophy or small vessel disease
Sulci And gyri	Evaluate each lobe separately, describing symmetry/asymmetry and comparing some reference areas (usually the occipital sulci and gyri) to the most reduced ones	Enlargement of sulci and reduced gyri not related to a specific cause (e.g., infarcts)
Medial temporal lobe	Evaluate each lobe separately, describing symmetry/asymmetry	Reduced hippocampal volume and enlarged widening temporal horn and choroid fissure
*Parenchymal signal changes*		
T2*/SWI changes	Evaluate number, size, symmetry topography, territory/pattern	Microbleeds (size < 10 mm), superficial siderosis, macrobleeds
		Lacunes (size: recent ≤ 20 mm; chronic 3–15 mm) and infarcts
FLAIR/T2/T1 weighted		Enlargement of perivascular spaces
		White matter changes (variable size, but > 1 mm)
Diffusion restriction		Recent infarcts and lacunes

**Table 3:** GCA-Koedam scale/score. GCA/Koedam score > 2 can be considered pathological

	Gyri	Sulci
0	Normal volume	Normal width
1 (considered normal in the elderly)	Normal	Slight opening of sulci
2	Reduced	Enlarged
3	Severely reduced (knife blade)	Severely enlarged

**Table 4:** Visual rating scale MTA-Scheltens

	Choroid fissure	Temporal horn	Hippocampal height
0	Normal	Normal	Normal
1	Widened	Normal	Normal
2	Moderately Widened	Widened	Reduced
3	Severely widened	Moderately widened	Moderately reduced
4	Severely widened	Severely widened	Severely reduced

Score based on the visual evaluation of the choroid fissures and temporal horns and hippocampi heights on a coronal T1 weighted image. The coronal section should be: (1) perpendicular to the long axis of both hippocampi, (2) symmetric, (3) placed at the level of the ventral pons or at the ponto-medullary sulcus. Each side should be rated and, in case of asymmetry, should be reported separately. Amygdala atrophy is part of this score and is performed by placing the previously described coronal section (1), (2), (3) anterior to the hippocampi

MTA = 2, bilaterally, or if > 2: Abnormal at all ages

MTA = 2, unilaterally: Abnormal in aged < 75 yrs

MTA < 2, normal

Global cerebral Atrophy (GCA) [49], Koedam for posterior lobes—which are most affected in atypical AD [34], MTA-Scheltens for medial temporal lobe [60].

**Fig. 5:**
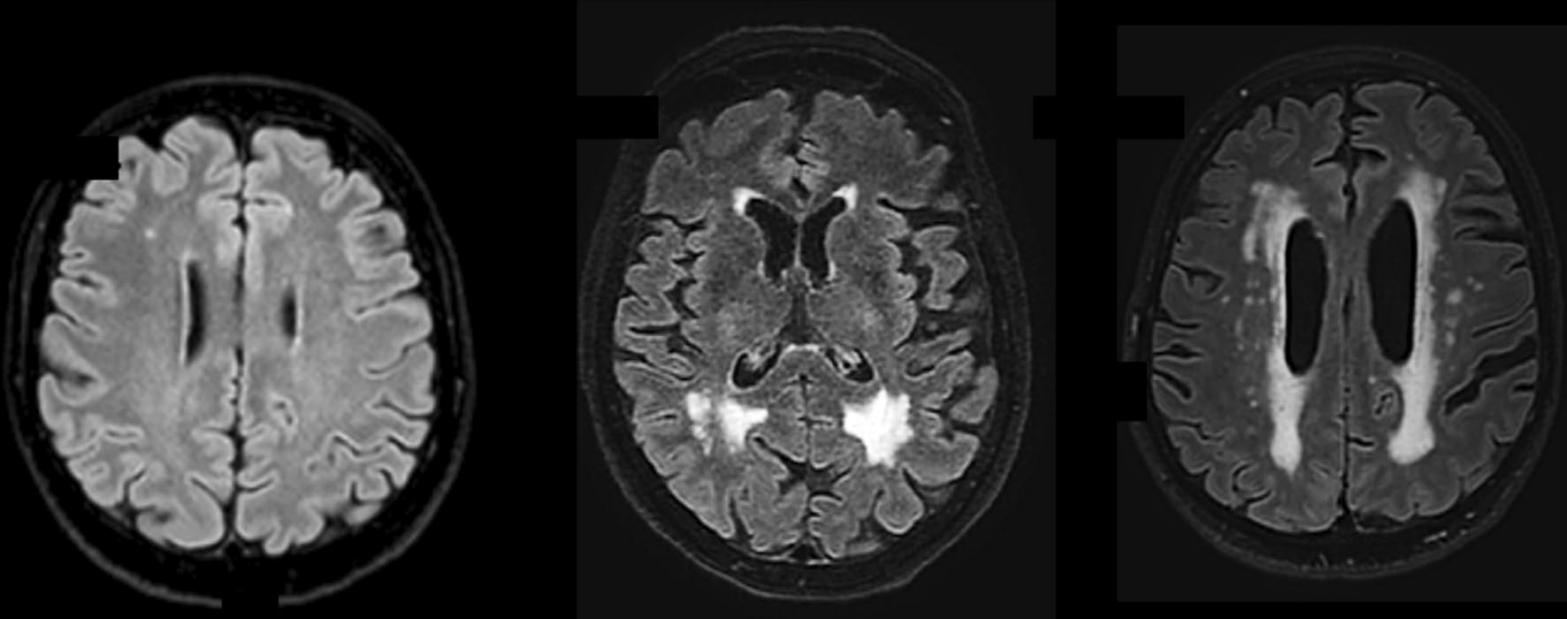
Axial FLAIR images. On the left, one punctate lesion on the right frontal white matter (Fazekas 1); in the middle, confluent foci at retrotrigonal white matter; on the right, diffuse and confluent subcortical, peri and paraventricular white matter lesions

**Fig. 6:**
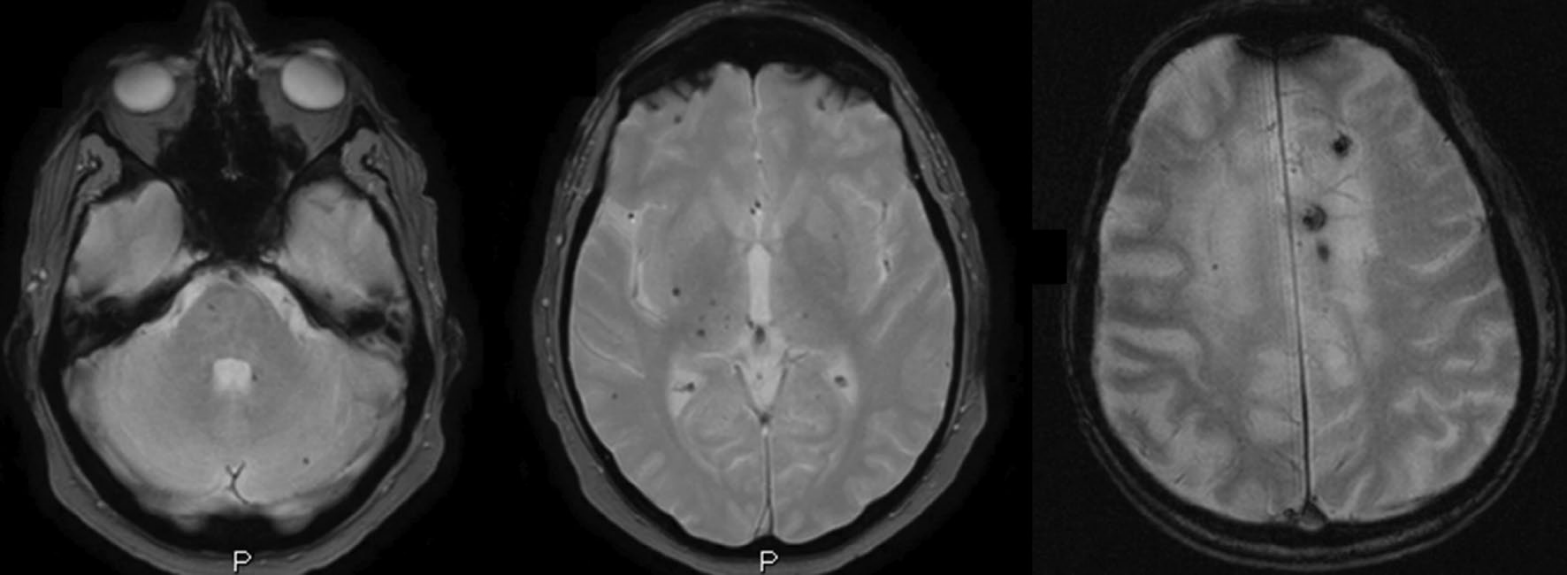
Axial GRE T2*. On the left image and middle images, infratentorial [pons (n. 1), left middle cerebellar peduncle (n. 1) and hemisphere (n. 1)] and deep [right thalamus (n. 5), posterior putamen (n. 1)] microbleeds, correlated to systemic hypertension. On the right image 3 lobar microbleeds associated with Fazekas 3, in keeping with amyloid angiopathy

(b) WHITE MATTER CHANGES (WMC) (see Tables 5 and 6; Fig. 5) should be evaluated in FLAIR/T2. The most used scales are Fazekas [17] and age-related white matter changes (ARWMC) [76]. The WMC have variable size, but minimal diameter of the lesions at imaging > 1 mm (in any plane).
(c) MICROBLEEDS should be evaluated in GRE T2*/SWI and the minimal diameter of the lesions at imaging is < 10 mm in any plane (Fig. 6). They could be a feature of small vessel disease (hypertension or cerebral amyloid angiopathy, CAA) and could be related to antithrombotic bleeding risk. All possible microbleeds mimics should be excluded (i.e., vessels, small cavernomas, mineralization foci, artifacts at the air-bone interface and due to partial volume, small hemorrhagic areas due to infarcts or other bleeds).

**Table 5:** Fazekas score

Fazekas
Punctiform or early confluent white matter lesions (Fazekas score 0–1) in periventricular or subcortical distribution is generally normal in aging
Fazekas score 2 can be considered normal in subjects of more than 70 years old
Confluent lesions (Fazekas score 3) indicate a risk of cognitive decline and physical impairment [69]

**Table 6:** ARWMC rating scale for WMLs on MR imaging and CT

*WMLs*	
0	No lesions (may include symmetric, well-defined caps or bands)
1	Focal lesions
2	Beginning confluence of lesions
3	Diffuse involvement of the entire region, with or without involvement of U-fibers
*Basal ganglia lesions*	
0	No lesions
1	1 focal lesion (3–5 mm)
2	> 1 focal lesion
3	Confluent lesions

Lesions are counted for the left and right hemispheres separately in these brain areas: frontal, parieto-occipital, temporal, infratentorial/cerebellum, and basal ganglia (striatum, globus pallidus, thalamus, internal/external capsule, and insula). For each of these regions, therefore, the sum score of the left and right hemispheres is from 0 to 6

An example of scale with high intrarater and interrater reliability is MARS (Microbleed Anatomical Rating Scale) which describes their number and location in lobar and/or infratentorial and/or deep regions [23].

**Fig. 7:**
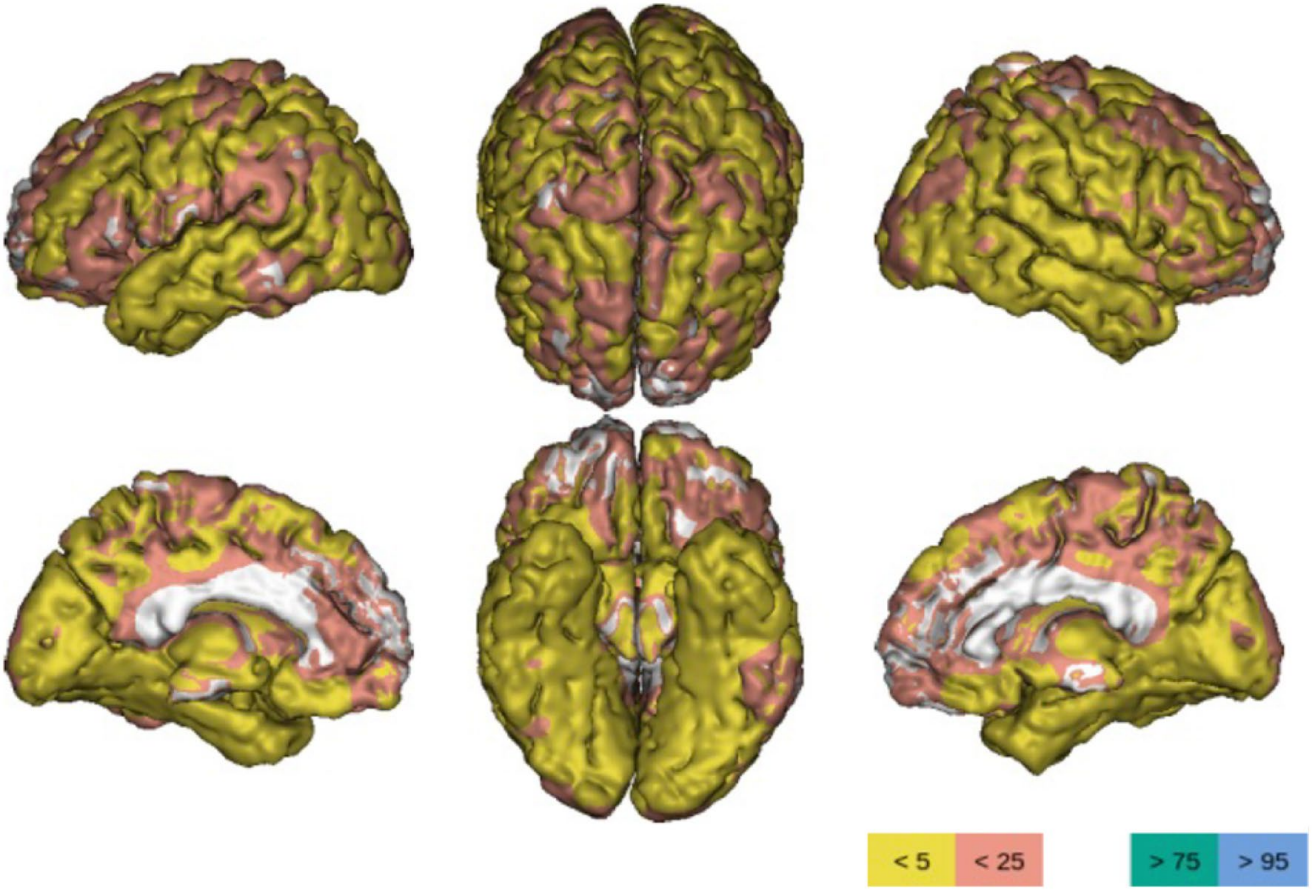
Three-dimensional brain rendering showing an example of quantitative analysis. Yellow and pink colors indicate the brain areas which are respectively below the 5 and 25 percentiles of the reference population (measures normalized to the intracranial volume). Powered by QyScore®

**Fig. 8:**
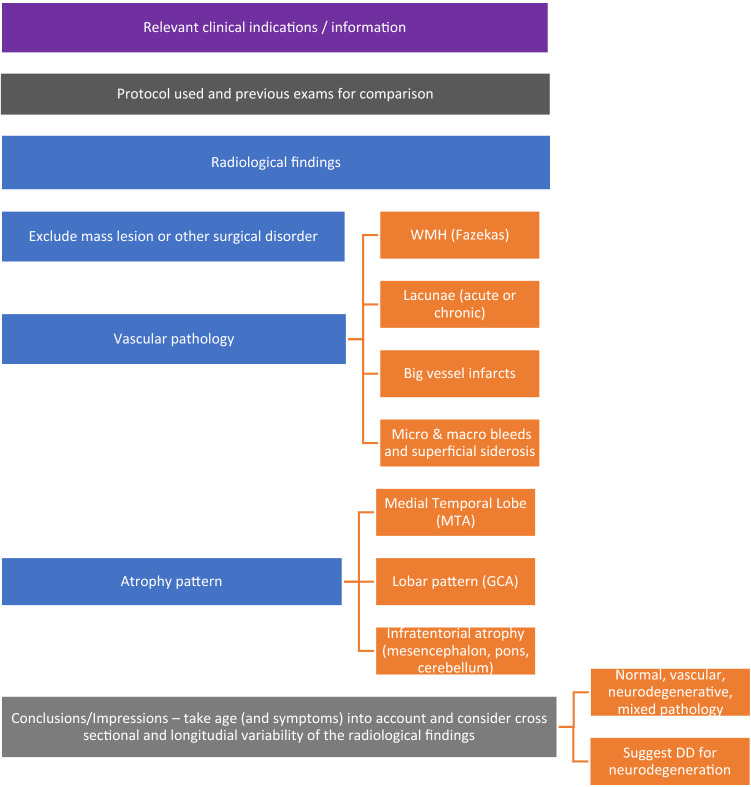
Guided report template

(d) SMALL VESSEL DISEASE (SVD) (see Figs. 5 and 6; Figs. 7 and 8) should be evaluated in FLAIR/T2, GRE T2*/SWI, and T1 acquisitions. Parenchymal changes such as (1) microbleeds; (2) lacunae; (3) perivascular spaces; and (4) white matter changes indicate the presence of small vessel disease, but each type of imaging finding has a different risk weight. In fact, the presence of a single lacuna or microbleed adds one point of the SVD score (Total 4 points) [69], which is equivalent to that of a severe enlargement of the perivascular spaces and/or Fazekas 3, thus indicating a higher risk of clinical consequence (ischemic and hemorrhagic brain events, dementia) [47].

3. Volumetric measures
  Visual differentiation between brain changes due to aging or to an early stage of the disease can be difficult, so the quantification of brain structures from a single patient and its comparison to age and sex-specific reference MRI data of healthy population can improve the diagnosis (Fig. 7). Several volumetric brain assessment methods and commercial Regulatory Authority approved (e.g., FDA, CE, CFDA marking) software are clinically available and implemented in radiology reporting, but without a clear strategy in the assessment. One way to improve the diagnostic accuracy of the use of the software in clinical practice is the double assessment—visual and quantitative—which combines the visual rating and the atrophy measurements [73].

4. Follow up
  If there are any vascular findings at MRI, an annual follow-up is recommended.
  In case of trials or other pathologies, the control MRI should be scheduled according to clinical indications.

**(D) Reporting**

1. A structured reporting is often not considered useful in clinical practice and could present other critical issues [74], so a guided report is preferable (Fig. 8). We recommend using the following template, modified from a previous ESNR dementia working group 2019 proposal.

Please consider that it is advisable to mention in the "Conclusions/Impressions" of the report:

- Any individual differences from a control population (cross-sectional assessment) by applying the visual qualitative and rating scale or/and the volumetric assessment
- Any stability or longitudinal worsening of the radiological findings (longitudinal evaluation) from previous radiological examinations, if appropriate for comparison

To do this, when evaluating brain atrophy, it is useful to take into account existing reference standards for assessing differences between a subject and the control population and individual rates of change over the life course even with respect to the trajectories of volumetric brain imaging markers [7, 75].

According to these large and inclusive datasets currently available (BrainChart open source and Rotterdam study), the trajectories of volumetric changes in gray matter, white matter, and third ventricles show nonlinear curves, with accelerated change with advancing age and some differences between men and women.

Regarding the "mixed pathology" reported in "Conclusions/Impressions," it should be emphasized that the diagnosis of "mixed dementia" is clinical, not neuroradiological. The neuroradiological description of, for example, hippocampal atrophy and Fazekas 3, does not mean that the patient's cognitive impairment is equally attributable to neurodegeneration and microangiopathy. It is up to the clinician to determine how much of the clinical picture is attributable to one or the other component.

### Sample Case Report (in Supplemental material)

**(E)** **Future avenues**

In the future, the use of volumetric information in routine radiology may be increasingly widespread, and we recommend dual assessment (combining visual scoring with volumetry, see C 3). These measurements are reproducible and automatic, but are depending on scan protocol, software, and the reference population. Other critical issues include the limited access to volumetric tools in the clinical setting (data must be transferred to the workstation and results to the PACS), and the training required to properly read the results.

Differences between men and women in neuroimaging biomarkers of neurodegeneration are reported [7, 75] and these should be considered in the near future when normative reference values will be applied in a clinical setting to assess pathology in individual patients.

The new Alzheimer drugs (i.e., Aducanumab) are rapidly changing the clinical scenario and the role of MRI, leading to the need for specific MRI protocols and precise reporting of the side effects of ARIA (amyloid-related imaging abnormalities, referring to cerebral edema or microhemorrhages).

One of the next frontiers is the clinical application of artificial intelligence, as it can offer solutions and interpretation of complex, multimodal medical information, such as that provided by imaging (radiology and nuclear medicine), biology, and neurocognitive testing, thus improving the diagnostic and prognostic process. But the process of identifying international medico-legal rules is still at an early stage [51].

## Discussion

To complement what was presented in the results, the main practical recommendation that emerged is to try to fit all the radiological steps presented—MRI protocol (B), image evaluation and interpretation (C), and reporting (D)—to the clinical diagnosis. Unfortunately, in radiological practice, there are still several general obstacles to this [74], such as:

(1) reduced confidence about the most correct approach to reading images (especially in the use of scales and volumetry), (2) report variability (with no use of structured or guided reports), and (3) generic requisition forms that do not allow radiologists to conclude whether imaging results are in line with clinical suspicion. The latter problem could be solved by better communication among specialists (e.g., at interdisciplinary meetings), which is essential in challenging clinical settings.

The use of all proposed scores is highly recommended, possibly accompanied by a visual description, except for MARS—which in routine is best replaced by a description of the number and distribution of microbleeds, according to the major patterns (superficial distribution in cerebral amyloid angiopathy versus deep infratentorial localization in hypertension)—and for the SVD score—which can be replaced by a description of the findings of small vessel disease according to the priority of their clinical relevance.

Although MRI findings are diagnostic only for a few conditions (e.g., late-onset AD, vascular dementia, CAA, iNPH, etc.), they support the clinical diagnosis of all forms of dementia (see *Boxes, above*) and provide important information on differential diagnosis, overlapping/coexisting forms (e.g., AD and VaD; FTLD and VaD; DLB and VaD), and possible side effects of new drugs.

More generally, neuroimaging is crucial for the diagnosis of dementia and is recommended in every patient with cognitive decline. In older adults, especially in the oldest old or in patients with multiple comorbidities, severe disability or behavioral disorders, completion of an MRI or nuclear imaging protocol can be troublesome, due to limited collaboration. The indications for the examination should be discussed with the treating physicians, ideally in a multidisciplinary team. Limited to these cases, volumetric CT is acceptable [2], at least to rule out some secondary and potentially reversible causes of cognitive impairment, such as subdural hematoma or brain masses.

## Conclusions

The diagnostic process of cognitive disorders requires a combined assessment of the clinical picture and imaging, including CT, MRI, and nuclear medicine, and can only be achieved through the dialogue between disciplines and the ongoing review of shared knowledge, information, and reports.

## Supplementary Information

Below is the link to the electronic supplementary material.

Supplementary file1 (DOCX 371 kb)

## Data Availability

Data sharing is not applicable to this article as no datasets were generated.

## Abbreviations

AD: Alzheimer disease
ADC: Apparent diffusion coefficient
AIDS: Acquired immunodeficiency syndrome
AINR: Italian Association of diagnostic and interventional NeuroRadiology (Associazione Italiana di Neuroradiologia Diagnostica ed Interventistica)
AIP: Italian Association of Psychogeriatrics (Associazione Italiana di Psicogeriatria)
aka: Also known as
ALS: Amyotrophic lateral sclerosis
APOE: Apolipoprotein E
APP: Amyloid precursor protein
ARIA: Amyloid related imaging abnormalities
ARWMC: Age-related white matter changes
ASL: Arterial spin labeling
AVIM: Asymptomatic ventriculomegaly with features of idiopathic normal pressure hydrocephalus on MRI
AVM: Arterio-venous malformations
bvFTD: Behavioral variant frontotemporal dementia
CA: Callosal angle
CAA: Cerebral amyloid angiopathy
CAA-ri: Cerebral amyloid angiopathy-related inflammation
CADASIL: Cerebral autosomal dominant arteriopathy with subcortical infarcts and leukoencephalopathy
CAPPAH: Convexity apparent hyperperfusion
CARASIL: Cerebral autosomal recessive arteriopathy with subcortical infarcts and leukoencephalopathy
CBD: Corticobasal degeneration
CBF: Cerebral blood flow
CBS: Corticobasal syndrome
CE: European community (comunità europea)
CFDA: China food and drugs administration
CJD: Creutzfeldt–Jakob disease
cMBS: Cortical microbleeds
CNS: Central nervous system
cSAH: Convexity subarachnoid hemorrhage
CSF: Cerebrospinal fluid
cSS: Cortical superficial siderosis
CT: Computed tomography
DESH: Disproportionately enlarged subarachnoid space hydrocephalus
DLB: Dementia with Lewy bodies
DWI: Diffusion weighted imaging
EMT: Endovascular mechanical thrombectomy
Eo-AD: Early-onset Alzheimer disease
ESNR: European society of neuroradiology—diagnostic and Interventional
FDA: Food and drug administration
FDG: Fluorodeoxyglucose
FLAIR: Fluid attenuated inversion recovery
FSE: Fast spin echo
FTD: Frontotemporal dementias
FTLD: Frontotemporal lobar degeneration
FU: Follow up
GCA: Global cerebral atrophy
gCJD: Genetic Creutzfeldt-Jakob disease
GRE: Gradient-recalled-echo
ICH: Intracerebral hemorrhage
iCJD: Iatrogenic Creutzfeldt-Jakob disease
iNPH: Idiopathic normal pressure hydrocephalus
IVT: Intravenous thrombolysis
KS: Korsakoff syndrome
LBD: Lewy body dementia
Lo-AD: Late-onset Alzheimer Disease
LOVA: Long-standing overt ventriculomegaly in adults
lvPPA: Logopenic variant primary progressive aphasia
MARS: Microbleed anatomical rating scale
MCA: Middle cerebral artery
MCI: Mild cognitive impairment
MND: Motor neuron disease
MRA: Magnetic resonance angiography
MRI: Magnetic resonance imaging
MTA: Medial temporal lobe atrophy
MTR: Magnetization transfer ratio
nfvPPA: Nonfluent/agrammatic variant primary progressive aphasia
NI: Neuroimaging
NPH: Normal pressure hydrocephalus
PACS: Picture archiving and communication system
PCA: Posterior cortical atrophy
PD: Parkinson's disease
PDD: Parkinson disease dementia
PET: Positron emission tomography
PiB: Pittsburgh compound B
PPA: Primary progressive aphasia
prnp: Prion protein
PRNP: Prion protein
PSEN1: Presenilin 1
PSEN2: Presenilin 2
PSP: Progressive supranuclear palsy
PVS: Perivascular spaces
REM: Rapid eye movement
sCJD: Sporadic Creutzfeldt–Jakob disease
SIGG: Italian Society of Geriatrics and Gerontology
SIRM: Italian Society of Medical and Interventional Radiology (Società Italiana di Radiologia Medica e Interventistica)
SPECT: Single-photon emission computerized tomography
SVD: Small vessel disease
svPPA: Semantic variant primary progressive aphasia
SW: Susceptibility weighted
SWI: Susceptibility weighted imaging
T: Tesla
TDP-43: Transactive response DNA-binding protein 43
TFNE: Transient focal neurologic episodes
TSE: Turbo spin echo
VaD: Vascular dementia
VCI: Vascular cognitive impairment
vCJD: Variant Creutzfeldt–Jakob disease
WE: Wernicke encephalopathy
WK: Wernicke–Korsakoff syndrome
WM: White matter
WMC: White matter changes
WMH: White matter hyperintensities
WML: White matter load
Yo: Years old
