# Supplementary material for: Radiological assessment of dementia: the Italian inter-society consensus for a practical and clinically oriented guide to image acquisition, evaluation, and reporting
Source: Radiol Med. 2022 Sep 7;127(9):998–1022. doi: 10.1007/s11547-022-01534-0 (PMC9508052; doi:10.1007/s11547-022-01534-0)
Supplement: Supplementary file 1 — Supplementary file1 (DOCX 371 kb) [file 11547_2022_1534_MOESM1_ESM.docx]

**Sample Case Report**

| Fig. a. Sample report. Top raw: Axial reconstructions of 3D FLAIR acquisition. Bottom: 2D axial T2* GRE and ADC map. |
| --- |
| 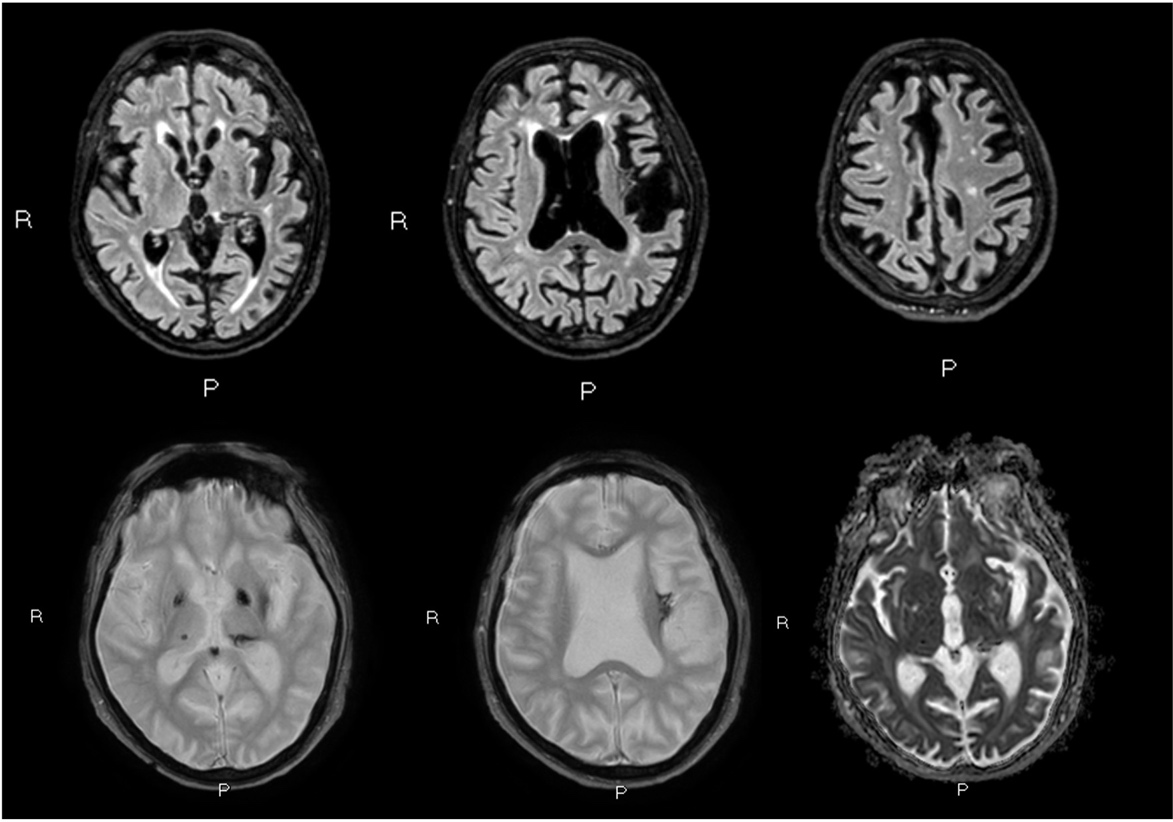 |

| Fig. b. Sample report. On the left, T1 multiplanar reconstruction (MPR) and, on the right, three-dimensional rendering of brain analysis with related percentile (Powered by QyScore®) |
| --- |
| 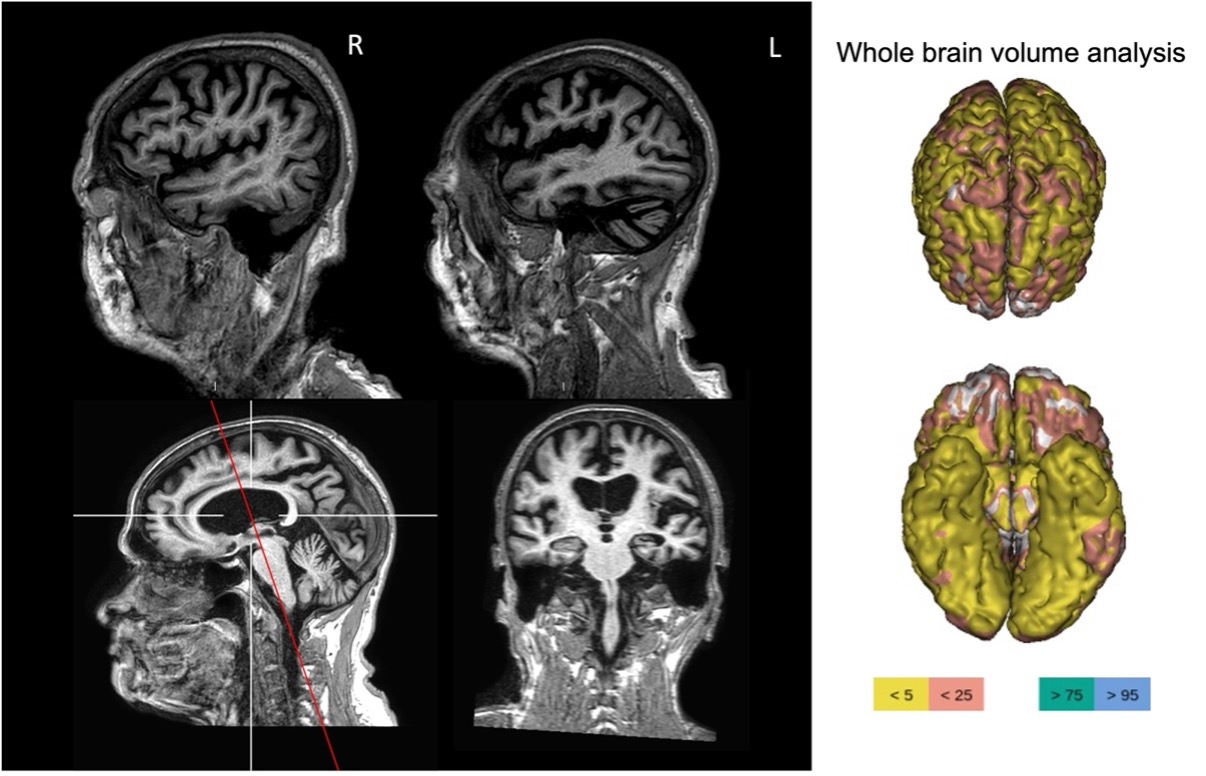 |

*Clinical information*: male, 65 yrs. old. Admission to Neurology for sub-entrant seizures and subsequent status epilepticus. History of alcohol abuse and cirrhotic hepatopathy.

*Technique*: volumetric T2, T1, FLAIR, axial GRE T2* at 1.5 Tesla MRI scan

*Findings*: Bilateral foci of signal hyperintensity on T2/FLAIR sequences at peri-paraventricular white matter, confluent at the watershed zones, in keeping with small vessel disease (Fazekas 2), with some subcortical lacunae.

Hemosiderin depositions are detected at the left insula (chronic haemorrhagic infarct) and at the level of the thalami (the larger one- on the left).

Lateral and third ventricles of enlarged volume. Fourth ventricle and cisterns within normal limits for patient’s age.

There is no midline shift.

Calcification of the pineal gland.

Moderate atrophy of the mammillary bodies and tectal plates.

Diffuse cortical CSF spaces enlargement (GCA=3 at the left insula/limbic lobe and MTA=4 on the left and MTA=2 on the right) (double assessment - visual and quantitative).

*Impressions / Conclusions:* moderate small vessel disease with lacunae, hemosiderin foci and chronic haemorrhagic infarct. Both visual evaluation and quantification are keeping with diffuse cortical atrophy, more severe on the left insular and limbic lobes (GCA=3) and on the left medial temporal lobe (MTA=4).

*Quantitative analysis: percentile range <5, 25, >75, >95. The individual subject's data is reported as abnormal if the volume or thickness of the cortex falls outside the 95% confidence interval. By 95% confidence interval, it means that if we measure the brain volume of 100 healthy people (i.e., free of neurological or psychiatric disease), the brain volume of 95 out of 100 people will fall within that range.
